# Effects of Mesenchymal Stem Cell Derivatives on Hematopoiesis and Hematopoietic Stem Cells

**DOI:** 10.15171/apb.2017.021

**Published:** 2017-06-30

**Authors:** Sara Aqmasheh, karim Shamsasanjan, Parvin Akbarzadehlaleh, Davod Pashoutan Sarvar, Hamze Timari

**Affiliations:** ^1^Stem Cell Research Center, Tabriz University of Medical Sciences, Tabriz, Iran.; ^2^Department of Pharmaceutical Biotechnology, Faculty of Pharmacy, Tabriz University of Medical Sciences, Tabriz, Iran.

**Keywords:** Hematopoietic Stem Cell, Cytokine, Mesenchymal Stem Cells, Microvesicle, miRNA

## Abstract

Hematopoiesis is a balance among quiescence, self-renewal, proliferation, and differentiation, which is believed to be firmly adjusted through interactions between hematopoietic stem and progenitor cells (HSPCs) with the microenvironment. This microenvironment is derived from a common progenitor of mesenchymal origin and its signals should be capable of regulating the cellular memory of transcriptional situation and lead to an exchange of stem cell genes expression. Mesenchymal stem cells (MSCs) have self-renewal and differentiation capacity into tissues of mesodermal origin, and these cells can support hematopoiesis through release various molecules that play a crucial role in migration, homing, self-renewal, proliferation, and differentiation of HSPCs. Studies on the effects of MSCs on HSPC differentiation can develop modern solutions in the treatment of patients with hematologic disorders for more effective Bone Marrow (BM) transplantation in the near future. However, considerable challenges remain on realization of how paracrine mechanisms of MSCs act on the target tissues, and how to design a therapeutic regimen with various paracrine factors in order to achieve optimal results for tissue conservation and regeneration. The aim of this review is to characterize and consider the related aspects of the ability of MSCs secretome in protection of hematopoiesis.

## Introduction

**Figure 1:**
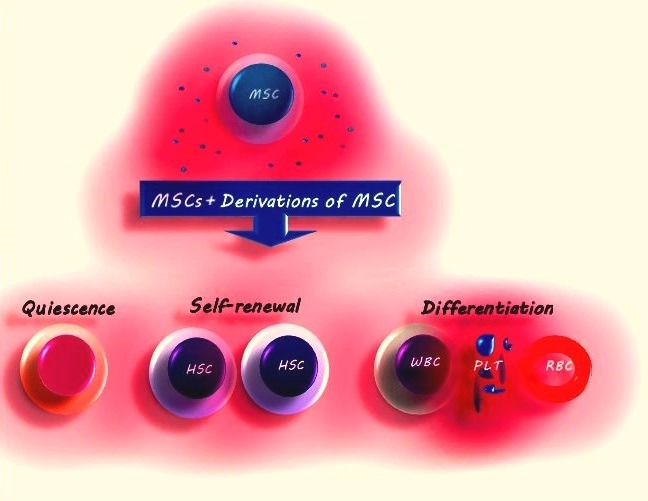
MSCs and their derivatives can regulate the action of HSPCs, such as self-renewal, differentiation, and quiescence

Hematopoiesis is a procedure in which hematopoietic stem and progenitor cell (HSPCs) show continued cellular actions, including self-renewal, apoptosis, proliferation, and differentiation into multiple lineages, which creates different types of mature blood cells, as well as sufficient numbers of blood cells required for maintaining homeostasis.^1^ This process is the result of cooperation between HSPCs and MSCs.^2^ Different HSPC subpopulations express the CD34 marker, which are the most undifferentiated stem cell type, as well as multipotent progenitors (MPPs) downstream of the differentiation hierarchy with capacity of multilineage production.^3^ Self-renewal is essential for maintaining the HSPC reconstitution and is therefore a prerequisite for lifelong hematopoiesis.^4^ Most HSPCs are quiescent and in G0 phase of cell cycle,^5,6^ and daily hematopoiesis is largely maintained by highly proliferative downstream HSPCs.^7^ Cellular actions of HSPCs are controlled by both intrinsic cellular factors such as transcriptional regulatory networks, as well as extrinsic cellular factors like growth factors, cytokines, chemokines and microvesicles (MVs); for example, G-CSF, CXCL12, and transforming growth factor-β (TGF-β).^8^ During embryonic, fetal, and adult life, hematopoiesis depends on a microenvironment involving soluble components and cell-cell interactions. This microenvironment is known as the hematopoietic niche, which is mostly derived from a common progenitor of mesenchymal origin that adjusts the steady HSPC quiescence and activation(Figure 1).^9^ Stem cells (including HSPCs or MSCs) assure the lifelong regeneration of tissues.^10^ Research has indicated that the cytokines and growth factors from MSCs exert their advantageous effects on target cells to boost tissue repair and regeneration, including immune response moderation, cell survival, anti-apoptosis, metabolism, proliferation, differentiation, hematopoiesis, angiogenesis, myogenesis, remodeling, wound healing, hair growth, neuroprotection, collateral development, and renal protection.^9,11-15^ The role of MSCs in support of hematopoiesis has been demonstrated by various studies. Dexter et al for the first time examined the establishment of *in vitro* culture conditions for long-term bone marrow culture (LTBMC) and showed that an adherent stromal-like culture could support the HSPCs.^16^ HSPCs are increasingly used for‏ allogeneic and autologous transplantation but recovery of platelets occurs with a lower rate; therefore, several studies have shown that the proliferation of HSPCs *in vitro* could result in faster recovery after transplantation.^17,18^ MSCs release many growth factors that stimulate hematopoiesis, prepare a scaffold for hematopoiesis, protect primitive progenitor cells, expand and maintain HSPCs in LTBMC with CD34 hematopoietic progenitor cells (HPCs), supporting both erythroid and myeloid differentiation.^19^

## Mesenchymal stem cells (MSCs)

Friedenstein was the first scientist who identified MSCs in bone marrow. He described an undifferentiated heterogeneous subset of‏ cells able to differentiate into mesenchymal lineages, such like osteocytes, adipocytes, and chondrocytes.^20,21^ MSCs can be isolated from various organs such as bone marrow, liver, adipose tissue, dental pulp, spleen, lung, umbilical cord blood,^22-24^ normal peripheral blood,^25^ and during or following normal pregnancy, with or without fetal origin.^26,27^ MSCs include 0.001%–0.01% of the nucleated cells in human bone marrow.^28^ The MSCs are largely believed to be derived from mesoderm; notably, the earliest lineage providing MSC-like cells during embryonic body formation is actually Sox1^+^ neuroepithelium rather than mesoderm, after which these early MSCs are replaced with MSCs from other sources in later processes.^29^ MSCs have been isolated from fetal blood, liver, and BM in the first-trimester of pregnancy with morphologic, immunophenotypic, and functional characteristics resembling adult-derived MSCs.^23^ Co-expression of surface markers and adhesion molecules like CD105 (SH2, transforming growth factor-b receptor III), CD73 (SH3&SH4, NT5E), CD90 (thy-1), CD29, CD44, CD106, CD166^30^ but lack of expression of hematopoietic stem cell markers CD34, CD45, CD117 (cKit), HLA class I, HLA-DR (except for HLA-ABC) and lineage-specific markers are important indicators of MSC immunophenotyping for detection of MSCs.^30-32^ MSCs have the ability of adhesion to plastic surfaces when cultured ex vivo with spindle-shaped and fibroblast-like morphology.^33^ MSCs can protect the reconstitution of erythroid, myeloid, lymphoid, and megakaryocytic lineages, which could improve hematopoietic engraftment.^34^ MSCs with immunosuppressive properties are useful in the treatment of graft versus host disease (GVHD)^35^ and can function through different ways from cell replacement to secretion of paracrine factors and cytokines.

## Hematopoiesis and Hematopoietic Stem and Progenitor Cells (HSPC)

Hematopoiesis is initiated by rare somatic multipotent‏ BM HSPCs and is a continuous process involving a hierarchy of differentiating progenitor cells, as well as production and consumption of mature blood cells that create the hemato-lymphoid system.^36^ HSPCs in the BM have two unique potentials: generating themselves (self-renewal capacity) and all other blood cells (multi-lineage differentiation capacity), i.e. erythrocytes, megakaryocytes/platelets, B/T lymphocytes, monocytes/macrophages, neutrophils/granulocytes, eosinophils and basophils, such that HSPCs proliferation is associated with their proliferation. The self-renewal capacity is necessary for homeostasis because mature blood cells have a short lifetime.^4^ HSPCs can be retrieved from BM, umbilical cord blood (UCB), and peripheral blood (PB) by apheresis after mobilizing HSPCs from BM to PB under the effect of granulocyte-colony stimulating factor (G-CSF). HPCs are uni-, bi-, or multi-potent, which have differentiation potential into various types of blood cells with limited self-renewal capacity.^37^ All functional HSPCs are associated with decreased and absence of expression of cell surface markers naturally detected on differentiating or mature blood cells while displaying Sca1 and c-kit markers. HSPCs can be identified with the absence of all lineage markers (Lin^−^) using a complex multi-flow cytometric labeling. CD34 is one of the most important markers, which is observed on early progenitor cells but not in mature cells, and CD38 is another surface marker that has been applied in association with CD34 to differentiate between HSPC, multipotent progenitors (CD38^−^), and committed progenitors (CD38^+^). Primitive HSPCs are CD34^+^,CD133^+^, CD38-, Lin^−^, Thy-1^+^(CD90), Sca1^+^, and c-kit^+^, while the coexpression of CD34^+^, CD38^−^, and CD90^−^defines MPPs. The expression of CD10 on CD34^+^ cells defines the lymphoid-committed progenitors and the expression of IL-3αR^lo^(CD123), CD45RA^−^ as well as CD34^+^ and CD38^+^ defines myeloid committed progenitors.^38,39^

Based on their self-renewal capacity, HSPCs are divided into two categories: LT-HSC (long term-HSC) with high self-renewal ability and ST-HSC (short term-HSC) with limited self-renewal power that are derived from LT-HSCs. ST- HSCs have the potential to differentiate to the common myeloid and lymphoid progenitors (CMP, CLP) and provide hematopoiesis for a short time.^21,40^ The promotion of cell differentiation is determined by increase in each of the CD13, CD38, CD45 and CD56 markers. Proliferation and differentiation of these cells are regulated by cell interactions, soluble components, intrinsic and extrinsic signals in embryonic yolk sac, placenta, liver, and finally in BM,^41-43^ respectively. Cell interactions are regulated by various extracellular matrix (ECM) proteins such as secreted growth factors, cytokines, adhesion molecules, MVs, transfer of genetic information,‏ and miRNA.^44-46^ ECM proteins, as well as MSCs, have effects on the maintenance and differentiation into lineage-committed HSPCs.^47^

## Potential signaling pathways associated with hematopoiesis

Signaling‏ pathways and cellular interactions adjust‏ the BM niche for HSPCs. MSCs produce numerous paracrine agents, and it may be difficult to investigate the mechanisms accountable for the production of distinctive factors.^48^ Some of these signaling pathways have been demonstrated to be associated with the expression and production of paracrine factors, involving a variety of signaling pathway receptors including Akt, signal transducer and activator of transcription (STAT), Tie2/Ang-1, p38 mitogen-activated‏ protein kinase (MAPK), and tumor necrosis factor (TNF). The study of Gnecchi et al demonstrated that MSCs express and produce paracrine factors that play a role in homing and reduction of apoptosis, including VEGF, FGF-2, Angiopoetin-1 (Ang-1), and hepatocyte growth factor (HGF) from MSCs. These are potential mediators of the impact of Akt-MSC conditional medium and are considerably up-regulated in the Akt-MSCs in response to hypoxia, representing that Akt signaling is critical to‏ the adjustment of the expression of these factors by MSCs.^49^ CCL5 (RANTES) and CXCL12 chemokines could activate STAT signaling pathways and are implicated in the survival and proliferation of HSPCs. CXCL12 selectively activates STAT-5 whereas CCL5 activates STAT-1, and these two chemokines also activate MAPK signaling pathways.^50^ HGFs can be divided into two types: upstream and downstream HGFs. The former induce HSPCs proliferation (most are asymmetric divisions), while the latter induce the committed progenitor cells to differentiate.^51^ The secretion of HGF, VEGF, and IGF-I by MCSs is crucially increased by stimulation with TNF, which is involved in the enhanced activation of p38 MAPK. Inhibition of p38 MAPK signaling significantly decreases the production of HGF, VEGF, and IGF-I. However, p38 MAPK inhibitor by itself has no influence on the production of these factors without TNF stimulation. Research shows that TNF promotes the production of paracrine factors in MSCs through a p38 MAPK-dependent mechanism.^52^ Also, the expression and production of CXCL-1, interleukin (IL)-6, and IL-8 is reduced through deactivation of p38 MAPK signaling in MSCs.^53^ p38 MAPKs are involved in the regulation of hematopoiesis, erythropoiesis, and myelopoiesis. p38MAPKs respond to different extracellular stimuli, especially cellular stress, including hypoxia, UV radiation, growth factors, and inflammatory cytokines^54^ and p38 activation can be induced by erythropoietin (EPO).^55^

Tie2/Ang-1 signaling pathway has a critical role in the maintenance of HSPCs. The tight adhesive binding of MSCs to HSPCs by Ang-1 ligand and tyrosine kinase receptor (Tie2) allows for a specific population of HSPCs to keep quiescence even in presence of mobilizing factors such as G-CSF, the stimulation of which is involved in the maintenance of LT-HSCs repopulating.^56^ Similarly, the thrombopoietin (TPO) receptor (c-Mpl) is expressed by a quiescent population of LT-HSCs that are found to be associated with TPO produced by MSCs, and the stimulation of this pathway increases the number of quiescent HSPCs, while its blockage leads to a reduction in LT-HSC.^57^

Through the production of Notch ligands via Wingless-type (Wnt) pathway, MSCs play a role in HSPCs^58^ survival and proliferation but inhibits their differentiation. In addition, through Jagged-1/Notch1,2 pathway, MSCs support HSPC self-renewal, which blocks differentiation into MPP and myeloid and monocytic cell lineage. Notch-1 promotes T-cell differentiation versus B-cell differentiation.^59,60^ Researchers have shown the expression of Notch-1 and Notch-2 by HSPCs, as well as Notch ligands Delta-1 (Dll-1) and‏ Jagged-1 (Jag1) by hMSC.^61^ Notch-1 plays an important role in the T- versus B-lineage selection of common lymphocyte precursors, but Notch-1 signaling has little role in the myeloid lineage differentiation.^62^ Further studies demonstrated that Notch-1 signaling increases the generation of precursor cells‏ and inhibits B-cell and myeloid differentiation, inducing T-cells so that the distinctive activation of Notch target genes results from selective activation of various Notch receptors as a result of specific ligand interactions, leading to diverse cellular outcomes.^63,64^ In addition, cross-talk between pathways such as the Notch and Wnt may lead to synergistic effects. Furthermore, soluble or cell-expressed Jagged-1 induced the expansion of HSPCs *in vitro* and mediated HSPC hematopoiesis and maintenance.^64,65^ Wnt/β-catenin signaling by MSC-MVs can improve the expansion of CD34^+^ cells^66^ through induced expression of the notch ligands (jagged-1, Dll-1)^67,68^ or p15^INK4b^ mRNA. Wnt pathway is involved in HSPCs self-renewal, proliferation, repopulating activity or lineage specific differentiation‏. Wnt pathway is activated by binding two types of receptors: the Frizzled family and a subset of low-density lipoprotein receptor-related protein (LRP) family (LRP-5 or 6). Since Wnt induces HSPC self-renewal in some organs, it enables the *in vitro* expansion of such cells and maintains their potency to reconstitute the entire cells after transplantation.^17,18^ Hedgehog signaling pathway modulates the transcription of target genes that affect the quiescence, self-renewal, proliferation, and differentiation of HSPCs. Three distinct ligands, i.e. Desert (Dhh), Indian (Ihh) and Sonic (Shh) Hedgehog exist in humans.

## MSCs derivatives

The MSCs represent important components of the microenvironment. They produce a large diversity of cytokines and soluble forms of adhesion molecules, e.g. vascular cell adhesion molecule-1 (VCAM-1) and intercellular adhesion molecule-1 (ICAM-1), which regulate hematopoiesis and are effective in homing similar to CXCL12.^69-71^ MSCs isolated from BM are functionally similar to umbilical cord blood derived MSCs^72^ and cytokine profile of BM and UCB MSCs is the same.^73^ A higher number of cytokines are released from placenta-MSC (P-MSCs) than umbilical cord-MSC (u-MSCs).^67^ The roles of some cytokines include maintaining HSPCs in quiescence, homing or induced self-renewal rather than differentiation. At the beginning of 1996, it was observed that MSCs isolated from human BM expressed and released G-CSF, stem cell factor (SCF), leukemia inhibitory factor (LIF), macrophage-CSF (M-CSF), IL-6, and IL-11 within the *in vitro* culture medium with a role in the adjustment of the differentiation of cells isolated from BM stroma through receptors related to gp130 and associated with signal transduction pathways.^69^ Most types of MSCs had a common expression pattern, including GRO-α (growth related oncogene α, CXCL1), IL-8 (CXCL8), and IL-6 that advance differentiation toward the myeloid lineage, as well as macrophage migration inhibitory factor (MIF, GIF, DER6) and Serpin E1 (PAI-1). Monocyte chemotactic protein-1 (MCP-1, CCL2) was expressed in both BM and amniotic MSCs, but the expression of stromal-derived factor-1 (SDF-1 or CXCL-12) involved in the homing and mediating the migration of HSPCs was higher in BM-MSCs.^74^ CCL2 acts as a strong chemotactic factor for monocytes, eosinophils, basophils, and a subset of T lymphocytes.^75^ Also, CXCL1, CXCL8, Serpin E1, and GM-CSF play a role in mobilization similar to G-CSF.^76^ A unique panel of chemokines, including CCR7, CCR9, CXCR4, CXCR5, and CXCR6 are involved in homeostatic leukocyte trafficking and cell compartmentalization within BM and/or in secondary lymphoid organs.^77,78^

The extracellular vesicles (EVs) derived from MSC are of three main types, including exosomes, microvescicles, and apoptotic bodies. They have different sizes (40–150 nm) and production mechanisms, and their cells of origin are determined by surface markers.^79-81^ These particles have a vital role in intercellular communication.^82^ MVs are derived both through outward budding surfaces of activated cells or follow the endosomal membrane formation after fusion of secretory granules with the plasma membrane, so that later exosomes are formed within the endosome and make multi-vesicular bodies (MVB)^83-85^ of varying size and composition. They often contain a number of factors, which include functional transmembrane proteins, cytoplasmic protein, bioactive lipids, messenger RNAs ‏)mRNAs‏(, tRNA, and microRNAs, mediating the transfer of these factors to target cells.^86^ Their RNA is nominated as “exosomal shuttle RNA” (esRNA). microRNAs (miRNA or miR) involve a class of small regulatory non-coding RNAs (19−23 nucleotides) that post-transcriptionally modulate gene expression, playing an important role in normal hematopoiesis by binding to their different target mRNAs.^87^ miRNAs have been implicated in all phases of hematopoiesis, including preservation of self-renewal and differentiation of HSPCs to mature blood cells, which might moderate cellular action by regulating transactivation, histone modification, DNA methylation, alternative splicing, and other miRNAs.^88,89^

The therapeutic effects of paracrine mechanisms of MSCs are extremely complex, including numerous cytokines, growth factors, as well as related receptors and signaling molecules with a wide area of biological functions.^27^ It is necessary to identify the factors involved in the adjustment of expression and production of these paracrine molecules in MSCs to gain an optimal therapeutic result.^90^ Effects of MSCs derivatives on HSPCs and hematopoiesis are summarized in Table 1.

## Regulation of HSPCs by MSCs derivatives

Signaling pathways associated with the maintenance and regulation of HSPCs obviously present useful knowledge on new findings in the treatment of various diseases and the developments in large scale preparation of HSPCs for transplantation.^39,53,56^ Also, the signaling pathways can provide understanding of the cancer stem cells to explore their possible use in treatments. All hematopoietic and immune cells are continuously generated by HSPCs through the intensely organized procedure of hierarchical lineage commitment.^4,39^ The MSCs represent important components with significant effects on different stages of hematopoiesis. Some of the cytokines released by MSCs are as follows: SCF, LIF, SDF-1, bone morphogenic protein (BMP)-4, Flt-3 ligand(FL), Kit-L, TNF-*α*, and TGF- *β*1.^69,83,84^ Some MSC cytokines can affect the maturation of HPCs, such as granulocyte-macrophage-CSF (GM-CSF), G-CSF, and also IL-1, IL-3, IL-6, IL-7, IL-11, IL-12, IL-14, IL-15 and TPO, as well as FL to promote self-renewal, proliferation, and differentiation of HSPCs.^84,91-93^ SCF, TPO, and FL are the most potent cytokines for HSPCs expansion. In contrast, IL-3, IL-6, IL-11, and G-CSF have a capacity to produce differentiated cells.^57^ A unique mix of immobilized ligand Delta1, fibronectin fragments, and cytokines (i.e. TPO, SCF,Flt3 ligand, IL-3, IL-6) led to increase in the number of CD34^+^ cells after 17 days of culture.^94^ TPO is important for early megakaryocyte differentiation and is modulated through c-mpl receptor and IL-11, resulting in platelet formation.^95^ IL-6 and G-CSF are necessary for myeloid differentiation, and IL-6 in combination with SCF can induce considerable proliferation of HSPCs.^96,97^ SCF/c-kit (CD117) in combination with FL/flt3 supports the preservation, proliferation, and differentiation toward myeloid and erythroid lineages of HSPC, as well as a number of other factors.^98-100^ BMP promotes blood production during *in vitro* differentiation. Kaimeng Hu et al showed that u-MSCs could be induced into hematopoietic cells and this differentiation is regulated through overexpression of *miR-218* and *miR-451* and affects the MITF-HoxB4 pathway.^101^*miR-451* is involved in specific differentiation of HSPC to erythroid lineage,^89^ as well as MV-mRNA that is involved in the hematopoietic differentiation along with Hexokinase 3 (HK3) and Eosinophil peroxidase (EPX).^102^* CEBPA/miR-182, EGR2/miR-150* and *miR-92, MPO/ hsa_piR_020814_ DQ598650* influence the down-regulated genes and therefore play a crucial role in cell death and differentiation.^88^ CEBPA-alpha regulates the equivalence between expansion and differentiation within early hematopoietic and myeloid development, which is controlled by *miR-182*.^103-105^ The *hsa_piR_020814_DQ598650* regulates myeloperoxidase (MPO) synthesis during myeloid differentiation.^106^*miR-150*^107^ and *miR-9*^108^ regulate another down-regulated gene, i.e. *Early Growth Response 2 (EGR2)*, which is involved in apoptosis and differentiation.^109,110^

MSCs also have a principal role in HSPC homing by secreting SDF-1,^111^ FL, SCF,^112^ VCAM-1, E-selectin, and collagen I,^113^ as well as expression of extracellular matrix proteins such as fibronectin, laminin, and vimentin in hematopoietic niche.^114-116^ The expression of SDF-1 chemokine is influenced by *miR-886-3p* that targets the 3´untranslated part of *SDF-1 mRNA*. SDF-1 plays a crucial role in early B-cell lymphopoiesis and hematopoietic regulation.^117,118^ HSPCs stick to fibronectin through at least two integrin pairs: VLA-4 (a4ß1) and VLA-5 (a5ß1). Fibronectin has either inhibitory or promotion effects on proliferation by inhibiting the G1/S promotion of HSPCs, which seems to be controversial.^119-121^ Laminin supports HSPCs proliferation and migration^122^ and chemokines conduct hematopoietic cell trafficking and localization in tissue.^123^ Several components can induce migration of HSPCs from BM to the peripheral blood. In the clinical setting, G-CSF is the most applicable inducer of HSPC mobilization,^124^ and *miR-126* in EVs of stem cells is required for the adjustment of HSPC mobilization by down-regulation of VCAM-1 on HSPC surface, causing a reduced mobilization response to G-CSF.^125^

In addition, chemokines released from MSCs such as CCL2, CCL5, CX3CL1 (fractalkine), CXCL8, CXCL12, and CXCL16 can stimulate chemotaxis.^126-128^ CXCL12 /CXCR4 protects the preservation, homing, quiescence, survival, and HSPCs development;^129-131^ also, G-CSF, VEGF, and CXCL16 are associated with HSPC homing.^132^ CX3CL1 protects cell growth, differentiation, and migration.^126^

BM-MSC-derived EV miRNAs can reduce apoptosis and differentiation of UCB-CD34^+^ cells.^88^ Overexpression of MV-miRs such as *miR221, miR451*, and *miR654-3p* induced cell development but the overexpression of *miR210-5p, miR106b-3p*, and *miR155-5p* inhibited radiation-induced apoptosis of HSPC.^133^ Luciana De Luca et al. demonstrated that BM-MSC-EVs can influence UCB-CD34^+^ gene expression model, resulting in the reduction of caspase dependent apoptosis via expression of *miR-21-5p, miR-181a-5p,* and* miR92a-3p*, inducing cell survival, inhibiting hematopoietic cell differentiation and boosting their movement to BM. Since these genes encode chemokines and cytokines (and their receptors) involved in the chemotaxis procedure of various BM cells, their potential role in the hematopoietic reconstitution is vital for engraftment.^88^* miR-223* has a role in HSPC proliferation.^134^ A study showed that *miR-223* was the highly expressed in platelets, peripheral blood mononuclear cells, and their plasma MVs.^135^

BM-MSC-EVs miRNAs/piRNAs such as *miR-21-5p*, *miR-181a-5p,* and *miR92a-3p *notably‏ reduce the apoptosis pathway and caspase 3/7 activity but *miR-27b-3p* and *miR-10a-5p* can reduce CD38 expression or gene expression pattern of up-regulated genes (for example, *IL6, CSF2, CCL3*) under the regulation of miRNA targeted genes (for example, *ZFP36/miR-27b-3p*).^88^

## Immunosuppressive Effects of MSCs Derivatives

MSCs have the immunosuppressive potential and can affect both natural and adaptive immunity by cell–cell contact or via secretion of soluble factors; however, the final effects depend on the type and condition of immune cells.^136^ Friedenstein showed that the transplantation of MSC/marrow stromal cells with HSPCs promotes the recovery of hematopoiesis and replicates the features of BM.^137^ Peng et al.‏ revealed that MSCs significantly increased the production of CD5^+^ regulatory B-cells via generation of IL-10.^138^ MSCs can inhibit DCs differentiation^139^ through the secretion of IL-6 and M-CSF and can eventually moderate immune responses via generation of growth factors and cytokines, including M-CSF, IL-6, prostaglandin E2 (PGE2), TGF-β, HGF, cyclooxygenase (COX)-1, COX-2, indoleamine 2,3-dioxygenase (IDO), nitric oxide (NO), and HLA-G5.^140-142^ PGE2 is capable of enhancing self-renewal and proliferation of HSPCs through interaction with Wnt pathway by elevating the β-catenin expression levels.^143,144^ Also, PGE2 affects macrophages so that MSCs may improve organ function and be effective in treating sepsis.^145^ TNF, IL-10, IL-6, and PGE2 inhibit DC maturation, T-cell function, as well as activation and proliferation of both B and NK cells.^146^ Moreover, HLA-G5 release by MSCs suppresses NK-cell and activity of T- and B-cells.^142^ IL-6 and the intercellular adhesion molecule 1 receptor inhibit T-cells, and have effects on B- cells.^147^ Increased IDO level is implicated in the differentiation of monocytes toward immune suppressive M2 macrophages, thus promoting the MSC immunosuppressive effect.^148^ In addition, MSC-EVs have the ability to suppress the maturation and activity of T- and B- cells, as well as differentiation of monocytes to M2-types, which is a result of functional development of CD4^+^,CD25^+^, highFoxP3^+^ regulatory T-cells (Tregs) through different ways, including CCL-1 induction and soluble HLA-G5 release.^27,142,149,150^ MSCs can inhibit T-cell proliferation, and activated T-cells are arrested in the G0 ⁄ G1 phase.^151,152^ MSCs express Toll-like receptors (TLRs) such as TLR3 and TLR4, which can inhibit the MSC immune-regulatory action by their ligands through Notch ⁄ Jagged1 signaling.^153^

**Table 1:** Effects of MSCs derivatives on HSPCs and Hematopoiesis

**MSCs derivatives**	**Effects on HSPCs and Hematopoiesis **	**References**
SCF TPO CXCL12 flt3l PGE2 miR221, miR451, miR654-3p	HSPCs expansion and development	^57,133^
IL-3, IL-6, IL-11 G-CSF	HSPCs differentiation	^57^
TPO	Early Megakaryocyte differentiation	^95^
IL-11	Platelet formation	^95^
IL-6 G-CSF	Myeloid differentiation	^96,97^
IL-6 in combination with SCF Laminin miR-223	HSPCs proliferation	^96,97,122,134^
SCF/c-kit (CD117)	Myeloid and Erythroid differentiation	^98,99^
CXCL8, CXCL12, CXCL16 CCL2, CCL5 CX3CL1 (fractalkine) Flt-3 ligand(FL) SCF, G-CSF, VEGF VCAM-1, E-selectin Collagen I Fibronectin, Laminin, Vimentin miR-126	HSPC homing and mobilization	^124-128^
CXCL12 (SDF-1)	Early B-cell lymphopoiesis Preservation, Quiescence	^117,118,129-131^
VEGF FGF-2 Angiopoetin-1 (Ang-1)	Homing and reduction apoptosis	^132^
IL-10	Increased CD5+ regulatory B cells generation	^138^
IL-6 and M-CSF	Inhibit DCs differentiation	^139^
M-CSF, HGF IL-6, IL-10 TNF PGE2 TGF-β COX-1, COX-2 IDO or NO HLA-G5	Immunosuppressive and immunomodulation	^140,141,146^
IDO	M2 macrophage differentiation	^148^
miR-451	Erythroid differentiation	^89^
CEBPA/miR-182 EGR2/miR-150 miR-92, miR-9, miR-150 MPO/ hsa_piR_020814_ DQ598650	Apoptosis and differentiation	^88,107,108^
hsa_piR_020814_DQ598650	Regulates MPO synthesis during myeloid differentiation	^106^
miR210-5p miR106b-3p miR155-5p	Inhibited radiation-induced apoptosis of HSPC	^133^
miR-21-5p miR-181a-5p miR92a-3p	Reduction of caspase dependent apoptosis	^88^

## Conclusion

BM has received special consideration because it contains MSCs as well as HSPCs. Utilization of MSCs provides for the regeneration of damaged organs with cell-cell contact, soluble factors, and autocrine or paracrine effects promoting their function and preparing considerable therapeutic advantages in different diseases^14,32^ through cell-free products from hMSCs that are effective on wound healing.^154^ *In vitro* expansion of HSPCs for transplantation is an intensive investigation field. The advantages of such investigations include accelerated engraftment, least stem cell harvests, reduced risk of infection, and enhanced effectiveness of genetically modified stem cells.^155^ The balance between self-renewal and proliferation of HSPCs will be helpful for the improvement of HSPC expansion and BM transplantation. MSCs and their derivatives have a critical role in homing, self-renewal, proliferation, and differentiation of HSPCs. Co-transplantation of MSCs and HSPCs promotes the engraftment of HSPCs and reduces the incidence of GVHD. This enhancement was higher after co-transplantation of HSPCs with GM-CSF and SCF-transfected MSCs, showing that these growth factors have effects on engraftment;^156,157^ therefore, these MSC cytokines and growth factors exert their advantageous effects on the target cells. Several studies have been conducted for demonstrating some of the effects of MSC on the expansion and differentiation of HSPC. Some studies on the expansion of HSPCs have been mentioned but further studies are required for the effects of MSCs on differentiation of HSPCs, especially the effects of MVs derived from MSCs, and the research for MSCs derivatives is an active subject of investigation. Novel and more sensitive devices and technology are required to discover, identify, and characterize recent MSCs derivatives that are found in low levels or have a labile nature.

## Ethical Issues

Not applicable.

## Conflict of Interest

The authors report no conflicts of interest.

## Abbreviations

MSC: Mesenchymal stem cell; HSPC: hematopoietic stem and progenitor cell; HSC: hematopoietic stem cell; MPP: multipotent progenitor; BM: bone marrow; HLA: human leukocyte antigen; GVHD: graft versus host disease; LT-HSC: long term-HSC; ST-HSC: short term-HSC; ECM: extracellular matrix; G-CSF: granulocyte-colony stimulating factor; TPO: Thrombopoietin; MAPK: mitogen-activated protein kinase; EVs: extracellular vesicles; MV: microvesicle; FL: Flt-3 ligand.
